# A new species of *Cisaris* (Hymenoptera, Ichneumonidae, Cryptinae) with a key to the world species

**DOI:** 10.3897/zookeys.136.757

**Published:** 2011-10-12

**Authors:** Shu-Ping Sun, Mao-Ling Sheng

**Affiliations:** General Station of Forest Pest Management, State Forestry Administration, Shenyang, Liaoning, 110034, China

**Keywords:** Phygadeuontini, *Cisaris*, new species, key, taxonomy, China

## Abstract

A new species, *Cisaris canaliculatus* Sun & Sheng, sp. n., belonging to the tribe Phygadeuontini of the subfamily Cryptinae (Hymenoptera: Ichneumonidae), collected from Jiangxi Province, China, is reported. A key to the species of the genus *Cisaris* Townes, 1970, is provided.

## Introduction

*Cisaris* Townes, 1970, belonging to the tribe Phygadeuontini of the subfamily Cryptinae (Hymenoptera, Ichneumonidae), comprises four described species (Yu et al. 2005), of which two are from the Oriental part of China (Kusigemati 1985, Pei and Sheng 2000, Sheng and Sun 2009), one from Japan (Kusigemati 1985), belonging to the Eastern Palaearctic Region, and one from the Philippines (Townes 1970). The status of the genus was defined by Townes (1970). There in an unknown number of undescribed species present in collection from the Oriental Region but few species occur in China or neighbouring countries.

In the last four years the authors have been exploring Jiangxi Province, situated in the northern border of the Oriental part of China. New discoveries have been reported (Sheng et al. 2009, 2010, 2011), and will be reported successively. In this article, one new species of *Cisaris* is reported.

## Materials and methods

Specimens were collected using entomological nets in the forests of Quannan, Yifeng and Zixi Counties, Jiangxi Province (CHINA). Images of whole bodies were taken using a CANON Power Shot A650 IS. Other images were taken using a Cool SNAP 3CCD attached to a Zeiss Discovery V8 Stereomicroscope and captured with QCapture Pro version 5.1.

The morphological terminology is mostly that of Gauld (1991). Wing vein nomenclature is based on Ross (1936) and the terminology on (Mason (1986, 1990).

Type specimens are deposited in the Insect Museum, General Station of Forest Pest Management, State Forestry Administration, People’s Republic of China.

## Taxonomy

### Cisaris

Townes, 1970

http://species-id.net/wiki/Cisaris

Cisaris Townes, 1970. Memoirs of the American Entomological Institute, 12(1969): 82. Type-species: *Cisaris tenuipe* Townes.

#### Diagnosis.

Head and mesosoma heavily punctate. Head comparatively large. Eye with sparse hairs. Margin of clypeus reflexed, median section often slightly produced. Mandible with upper tooth much longer than lower tooth. Notaulus not reaching to center of mesoscutum. Posterior edge of mesoscutum without transverse groove. Scutoscutellar groove without median longitudinal carina. Fore wing without areolet. Fore wing vein 2m-cu subvertical, with one bulla. Area superomedia hexagonal or trapezoidal. Hind tibia with dense inner, apical fringe of setae and with polished groove between setal fringe and tarsal insertion. First to third terga polished. First tergum slender, spiracle far behind middle. First sternite slightly basal of spiracle. Ovipositor compressed, its tip very long and gradually tapered. Dorsal valve with a weak nodus. Ventral valve without ridges.

#### Key to the species of *Cisaris*

**Table:** 

1	Female	2
–	Male	6
2	Antenna without white ring. Fore wing with vein 1cu-a distad of 1/M. Area superomedia longer than wide, with costula near its middle. (Male unknown)	*Cisaris tenuipes* Townes
–	Antenna witht white ring. Fore wing with vein 1cu-a opposite 1/M. Area superomedia wider than long, or approximately as long as wide, with costula behind its middle or near its posterior corner	3
3	Terga red or reddish brown. Area superomedia and area petiolaris separated by a strong carina	4
–	Terga black. If black with apical terga blackish brown (Fig. 1), area superomedia and area petiolaris combined (Fig. 5)	5
4	Face 1.8 to 1.9 times as wide as long at level of upper margin. Malar space approximately 0.6 times as long as basal width of mandible. Costula originating from anterior corner of area petiolaris	*Cisaris takagii* Kusigemati
–	Face 2.2 times as wide as long at level of upper margin. Malar space approximately 0.92 times as long as basal width of mandible. Area superomedia with costula slightly behind its middle	*Cisaris mitis* Pei & Sheng
5	Area superomedia trapezoidal, anterior and posterior sides (carinae) very weak or almost absent, with costula at its posterior corner, transverse (Fig. 5). Ocular-ocellar line at least 2.0 times as long as largest diameter of ocellus (Fig. 3)	*Cisaris canaliculatus* Sun & Sheng, sp. n.
–	Area superomedia hexagonal, with complete and strong carinae, costula originating from its posterior corner, leaning slightly forward laterally. Ocular-ocellar line 1.6 times as long as largest diameter of ocellus. (Male unknown)	*Cisaris niger* Kusigemati
6	Terga and hind leg entirely black (Fig. 2). Malar space 0.42 to 0.47 times as long as basal width of mandible. Area superomedia and area petiolaris completely combined	*Cisaris canaliculatus* Sun & Sheng, sp. n.
–	Terga brown or darkish brown. Hind leg reddish brown or darkish brown. Malar space at most 0.3 times as long as basal width of mandible. Area superomedia separated from area petiolaris by strong carina	7
7	Area superomedia approximately 1.5 times as wide as long, costula originating from its middle	*Cisaris mitis* Pei & Sheng
–	Area superomedia approximately 2.7 times as wide as long. Costula originating from anterior corner of area petiolaris	*Cisaris takagii* Kusigemati

### Cisaris canaliculatus

Sun & Sheng sp. n.

urn:lsid:zoobank.org:act:0836E352-AA13-4BC0-8509-7317277E2D0D

http://species-id.net/wiki/Cisaris_canaliculatus

**Figure 1.**
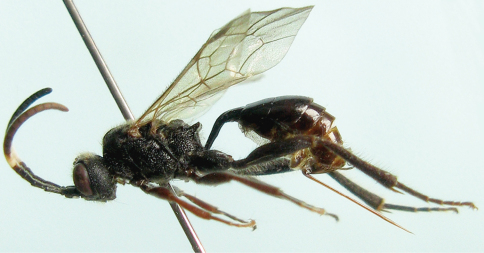
*Cisaris canaliculatus* Sun & Sheng, sp. n. Female (Holotype). Body, lateral view.

Figures 1 [Fig F2] [Fig F3] [Fig F4] [Fig F5] 6

#### Etymology.

The specific name is derived from the median trough of the propodeum.

#### Material examined.

*Holotype*: female. CHINA: Matoushan, 400m, Zixi County, Jiangxi Province, 8 May 2009, leg. Mei-Juan Lou. *Paratypes*: 1 female, CHINA: Quannan County, Jiangxi Province, 29 April 2008, leg. Shi-Chang Li; 15 males, same data as holotype except 10 to 17 April 2009; 1 male, CHINA: Guanshan Natural Reserve, Yifeng County, Jiangxi Province, 20 April 2009, leg. Mao-Ling Sheng; 2 males, CHINA: Guanshan Natural Reserve, Yifeng County, Jiangxi Province, 24 April 2011, leg. Shu-Ping Sun and Mao-Ling Sheng.

#### Diagnosis.

Head, mesosoma, coxae and terga black, except apical portion of female more or less blackish brown. Flagellum with white ring. Tegula brownish black. Area superomedia trapezoid, anterior and posterior sides of area superomedia very weak or almost absent (Fig. 5). Apical-median portion from area superomedia to apex of area petiolaris strongly concave longitudinally. Costula present at posterior corner of area superomedia. Propodeal apophysis very strong and compressed.

#### Description.

Female. Body length 7.5 to 8.0 mm. Fore wing length 6.5 to 7.0 mm. Ovipositor sheath length 2.0 to 2.5 mm.

Head. With dense brown hairs. Face very wide, 2.4 to 2.5 times as wide as long at level of upper margin, distinctly convex centrally, with dense and uneven punctures, near upper margin, beneath antennal socket, with shallow transverse groove. Lateral portion of clypeal suture deep, median portion weak. Clypeus evenly convex, 2.5 to 2.6 times as wide as long; with punctures as that of face; subapical margin distinctly raised, slightly concave centrally; apical margin weakly cambered forward. Mandible strong, upper and lower margins almost parallel, basal portion with dense punctures and brown hairs, apical portion smooth. Upper tooth of mandible sharp, 1.7 to 1.8 times as long as lower tooth. Cheek with coarse punctures. Malar space approximately 0.8 times as long as basal width of mandible. Subocular sulcus vestigial. Gena wide, slightly convergent backwardly, in dorsal view 1.0 to 1.1 times as long as width of eye, with dense punctures, distance between punctures 0.2 to 1.5 times diameter of puncture. Vertex (Fig. 3) not convex, between eye and lateral ocellus with correspondingly sparse and irregular punctures, distance between punctures 0.5 to 2.5 times diameter of puncture; on the portion between lateral ocelli and occipital carina with punctures as that of gena. Postocellar line about 0.6 times as long as ocular-ocellar line. Ocular-ocellar line 1.9 to 2.0 times as long as largest diameter of ocellus, 2.2 to 2.3 times as long as shortest diameter of ocellus. Frons approximately flat, with dense and irregular punctures, distance between punctures 0.2 to 0.5 times diameter of puncture. From fifth flagellomere to apex of antenna correspondingly thicker than basal four flagellomeres, scape almost cylindric, apical truncation weakly oblique, approximately 15 to 16 degrees from transverse; with 17 flagellomeres, slightly thickened beyond middle. Ratio of length from first to fifth flagellomeres: 2.6:2.5:2.0:1.4:1.2. Occipital carina complete and strong.

Mesosoma. With dense brown hairs. Pronotum with dense and irregular punctures, anterior portion rough, with indistinct longitudinal wrinkles, dorsal-anterior portion with distinct short longitudinal wrinkles, dorsal-porsterior smooth and shining. Epomia strong. Mesoscutum with dense punctures, distance between punctures on anterior and lateral portion 0.2 to 0.5 times diameter of puncture; posterior-median portion slightly rough, punctures elongate. Notauli present, anterior 0.3 sharp. Posterior edge of mesoscutum distinct, without transverse groove. Scutoscutellar groove deep, almost “U-shaped”, with dense longitudinal wrinkles. Scutellum almost flat, with dense and irregular punctures. Postscutellum rough, small, rectangular, anterior-lateral portion deeply concave. Mesopleuron (Fig. 4) extremely rough, with dense and irregular wrinkles and indistinct punctures, median portion with indistinct transverse wrinkles. Subalar prominence convex, as a thin lobe. Epicnemium with short transverse carina opposite lower corner of pronotum. Epicnemial carina strong, upper end reaching to subalar prominence. Speculum small and smooth, or with punctures. Mesopleural fovea consisting of a deep horizontal groove that connecting with mesopleural suture. Anterior half of sternaulus deep, posterior half weak, reaching to posterior margin of mesopleuron above its lower posterior corner. Metapleuron coarse, with reticulate wrinkles. Juxtacoxal carina complete. Submetapleural carina strongly lobed. Legs with dense brown hairs. Hind coxa and outer profile of hind femur with dense punctures. Hind tibia coarsely sculptured. Ratio of length of hind tarsomeres 1:2:3:4:5 is 5.2:2.0:1.6:1.0:1.6. Wings slightly brownish, hyaline. Fore wing with vein 1cu-a opposite 1/M. Vein 2rs-m approximately as long as distance between it and 2m-cu. Vein 2-Cu approximately 2.0 times as long as 2cu-a. Hind wing M+Cu slightly arched. Vein 1-cu distinctly inclivous, about 2.0 times as long as cu-a. Propodeum (Fig. 5) with strong carinae, punctures large and dense, lateral and apical portion with reticulate texture. Area basalis short and wide. Area superomedia trapezoid, slightly wider than long. Median sections of anterior and posterior transverse carinae, anterior and posterior sides of area superomedia, very weak or almost absent. Apical-median portion from area superomedia to apex of area petiolaris strongly concave longitudinally. Costula present at posterior corner of area superomedia. Propodeal apophysis very strong and compressed. Propodeal spiracle almost round.

Metasoma. Terga smooth and shining, apical portion compressed. First and second terga without punctures. First tergum slender, 2.4 to 2.5 times as long as apical width. Median dorsal carina weak, reaching to spiracle. Dorsolateral carina weak but complete. Spiracle small, round, beneath dorsolateral carina, slightly convex, located approximately at apical 0.3 of first tergum. Second tergum about 0.7 to 0.8 times as long as apical width. Third tergum about 0.7 times as long as basal width, lateral portion and posterior margin with sparse and fine punctures and weak brown hairs. Posterior portions of remaining terga with distinct and fine punctures and brown hairs. Ovipositor sheath about 0.95 to 1.0 times as long as hind tibia. Nodus of dorsal valve indistinct.

Color (Fig. 1). Black, except the following. Flagellomeres 5 to 7 (8) white, ventral profile of apical flagellum taupe. Apical portion of mandible except black teeth reddish brown. Front and mid coxae, trochanters and basal portions (without dividing line and gradually changed to apical portion) of femora brownish black; apical portions of femora, tibiae and tarsi reddish to darkish brown. Apical portion of metasoma obscurely blackish brown. Maxillary and labial palpi yellowish brown, tegula brownish black. Fore wing veins and stigma brownish black. Hind wing veins brownish yellow.

**Figure 2. F2:**
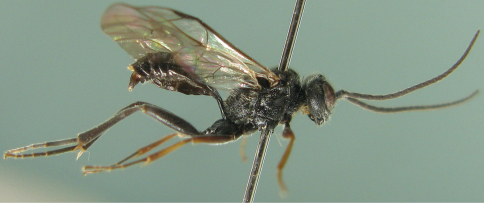
*Cisaris canaliculatus* Sun & Sheng, sp. n. Male. Body, lateral view

Male (Figs. 2, 6). Body length 6.5 to 8.0 mm. Fore wing length 6.0 to 7.0 mm. Head correspondingly large. Malar space approximately 0.42 to 0.47 times as long as basal width of mandible. Antenna slightly shorter than body, with 22 flagellomeres, without white ring. Lateral-median portion of pronotum, beneath epomia, glazed and shining. Median portion of mesopleuron (Fig. 6) coarse and with distinct oblique transverse wrinkles, subupper portion transversely smooth. Area basalis very short and wide. Area superomedia and area petiolaris completely combined, straight slanted from anterior transverse carina to apex of propodeum. Front and middle coxae, trochanters, hind legs, tegula and all terga black. Other characters as in female.

#### Variation.

The sculpture of the male mesopleuron varies from distinct oblique transverse wrinkles or almost without wrinkles and with distinct punctures.

#### Remarks.

Similar to *Cisaris niger* Kusigemati 1985, but can be easily distinguished from the latter in having the pronotum with a smooth, shining and impunctate area on the subdorsal portion; the propodeal apophysis (Figs 1, 5) is very strong and compressed; the area superomedia and area petiolaris are combined. In *Cisaris niger* the pronotum lacks an impunctate area on the subdorsal portion; the propodeal apophysis is indistinct; the area superomedia and area petiolaris are separated by a strong carina. It can be separated from all other known species by the key.

**Figure 3. F3:**
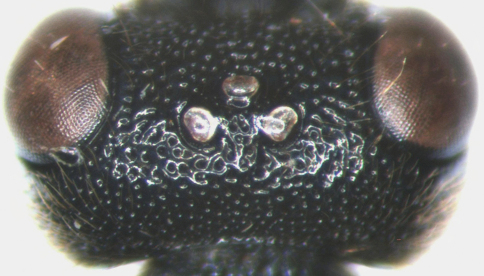
*Cisaris canaliculatus* Sun & Sheng, sp. n. Female (Holotype). Vertex.

**Figure 4. F4:**
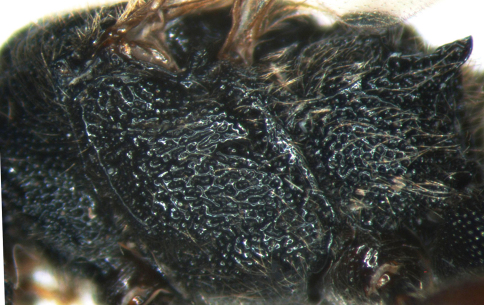
*Cisaris canaliculatus* Sun & Sheng, sp. n. Female (Holotype). Mesopleuron.

**Figure 5. F5:**
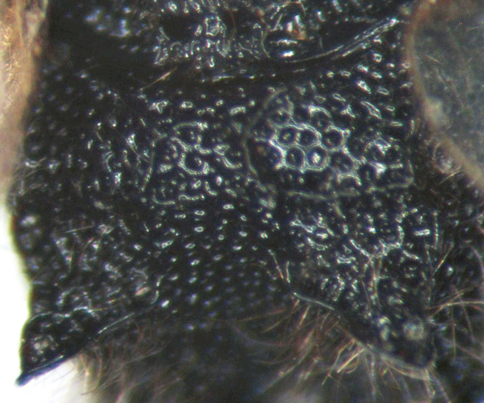
*Cisaris canaliculatus* Sun & Sheng, sp. n. Female (Holotype). Propodeum.

**Figure 6.**
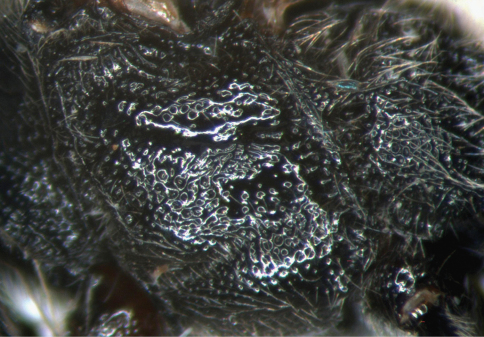
*Cisaris canaliculatus* Sun & Sheng, sp. n. Male. Mesopleuron.

## Supplementary Material

XML Treatment for Cisaris

XML Treatment for Cisaris canaliculatus
